# Amiodarone‐Induced Pulmonary Toxicity in an Elderly Patient: A Case Report

**DOI:** 10.1002/ccr3.71245

**Published:** 2025-10-10

**Authors:** Bal Krishna Subedi, Naveen Gautam, Anuja Upadhyay, Parikshit Chapagain, Daniel Bitetto

**Affiliations:** ^1^ Department of Internal Medicine Jefferson Einstein Montgomery Hospital East Norriton Pennsylvania USA; ^2^ Department of General Medicine Gulmi Durbar Basic Hospital Gulmi Nepal; ^3^ Department of Internal Medicine AdventHealth Orlando Orlando Florida USA

**Keywords:** adverse drug reaction, amiodarone, amiodarone pulmonary toxicity, atrial fibrillation, case report, elderly patient

## Abstract

Amiodarone, a widely utilized and effective class III antiarrhythmic agent, carries a significant risk of severe organ toxicities, notably including the pulmonary system. This report details a case of an 82‐year‐old female who developed amiodarone‐induced pulmonary toxicity (APT) following 2 years of standard 200 mg daily maintenance therapy for atrial fibrillation. The patient presented with progressive dyspnea, non‐productive cough, and new‐onset oxygen dependency. Diagnostic evaluation, including chest computed tomography revealing diffuse interstitial opacities, alongside the systematic exclusion of other etiologies, confirmed the diagnosis. Prompt discontinuation of amiodarone and initiation of corticosteroid therapy resulted in clinical stabilization. This case underscores the insidious nature of amiodarone‐induced adverse effects, particularly in the elderly population, and reinforces the critical necessity for a high index of clinical suspicion and vigilant, proactive monitoring strategies to facilitate early detection and appropriate management of this potentially life‐threatening iatrogenic complication, even with standard therapeutic dosages.

**Summary**

- Amiodarone, though effective for arrhythmias, may cause insidious pulmonary toxicity even at standard doses in older adults.
- This case highlights the need for baseline DLCO, vigilant monitoring, early imaging when symptoms arise, and prompt discontinuation with steroids when indicated to prevent irreversible lung damage.

## 1 Introduction

Amiodarone is a potent class III antiarrhythmic medication widely utilized for managing cardiac dysrhythmias, including ventricular arrhythmias and atrial fibrillation [1, 2]. Despite its efficacy, amiodarone exhibits complex pharmacokinetics characterized by variable absorption (22%–86%), extensive tissue distribution, and an exceptionally long elimination half‐life (25–107 days) [3, 4]. These properties contribute to its persistent effects for months after discontinuation and necessitate careful monitoring during long‐term administration [3]. Among amiodarone's numerous adverse effects, amiodarone‐induced pulmonary toxicity (APT) represents one of the most serious complications, with reported incidence ranging from 1.6% to 2% with lower doses to 5%–15% with higher doses [5, 6]. Risk factors for APT include cumulative dose, treatment duration exceeding 2 months at ≥ 400 mg/day or 2 years at 200 mg/day, age over 60 years, and pre‐existing lung disease [5, 7]. The pathogenesis involves direct cytotoxic effects through phospholipid accumulation and indirect immune‐mediated inflammatory responses [8]. APT can manifest as interstitial pneumonitis, organizing pneumonia, acute respiratory distress syndrome, diffuse alveolar hemorrhage, or pulmonary nodules [5, 8]. This case is unique as it illustrates amiodarone‐induced pulmonary toxicity in an elderly female with obstructive sleep apnea and a remote history of breast cancer, conditions that could confound the diagnosis. The report emphasizes distinguishing APT from alternative etiologies and underlines practical monitoring lessons for clinicians.

## 2 Case Presentation

An 82‐year‐old female presented with progressively worsening shortness of breath over a two‐week period, exacerbated by physical activity and improved by upright position. The clinical course is summarized chronologically: (1) 2 weeks before admission‐ viral‐like illness with cough, chills, and fever; (2) 1 week before admission‐ worsening dyspnea and wheeze; (3) presentation‐ progressive hypoxemia requiring oxygen. The symptom had been preceded by a possible viral illness 2 weeks prior. She had experienced dry cough with chills and low‐grade fever, accompanied by shortness of breath and wheezing that partially improved but never fully resolved. One week prior to presentation, she noted worsening wheezing, shortness of breath, and dry cough. The patient was a lifelong non‐smoker without environmental or occupational exposures. Vital signs showed blood pressure 166/68 mmHg, respiratory rate 26 breaths/minute, heart rate 68 bpm, temperature 36.6°C, and oxygen saturation 78% requiring 4–6 L/min supplementation. BMI was 38 kg/m^2^ with body weight 95 kg. Examination revealed labored respirations, bilaterally reduced breath sounds, and rales in lower lung fields without wheezing.

### 2.1 Past Medical History

Medical history included atrial fibrillation treated with apixaban and amiodarone (200 mg daily for approximately 2 years prior to presentation), prediabetes, hypertension, hyperlipidemia, degenerative joint disease, insomnia, and recently diagnosed obstructive sleep apnea on CPAP therapy. Remote history of breast cancer treated with radiation and chemotherapy more than 20 years prior. Pulmonary function tests (PFTs) performed after the initiation of amiodarone showed a diffusing capacity for carbon monoxide (DLCO) of 13.59 mL/min/mmHg (74% of predicted). The patient denied prior COPD, asthma, or interstitial lung disease other than the findings related to the current presentation.

### 2.2 Medication History

At the time of presentation, the patient's medication regimen included amiodarone 200 mg daily for atrial fibrillation, for which she was also taking apixaban 5 mg twice daily. Her antihypertensive medications included nebivolol 10 mg daily, amlodipine 5 mg daily, hydralazine 25 mg twice daily, olmesartan 20 mg daily, and spironolactone 25 mg daily. Other medications were escitalopram 10 mg daily, zolpidem 5 mg at bedtime for insomnia, and rosuvastatin 10 mg daily for hyperlipidemia.

### 2.3 Laboratory and Imaging Findings

**FIGURE 1:**
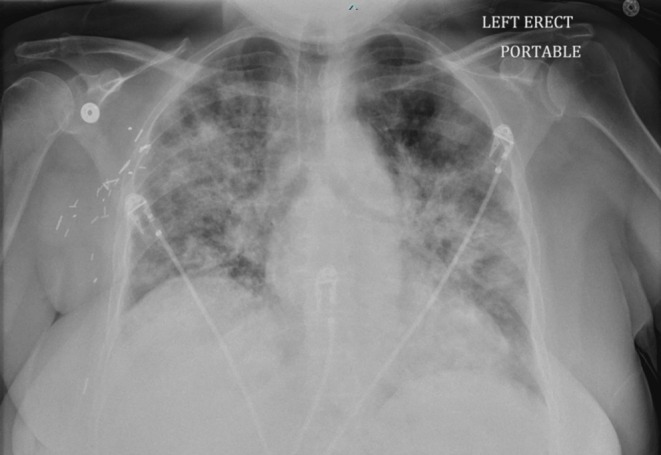
Initial chest radiograph showing diffuse interstitial and alveolar opacities bilaterally.

**FIGURE 2:**
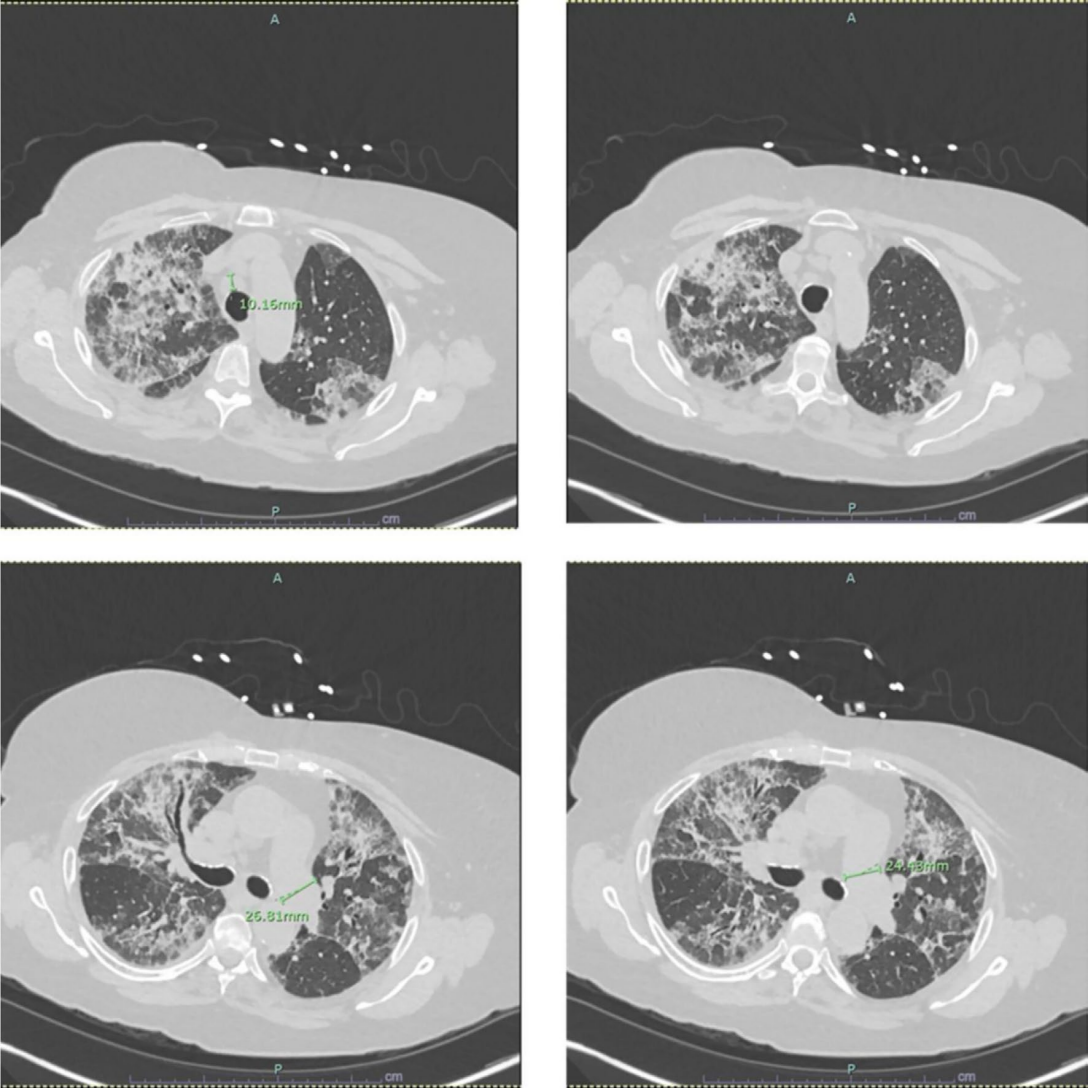
Non‐contrast Chest CT showing diffuse interstitial and alveolar opacities with upper lobe predominance.

Laboratory investigations revealed several notable findings, detailed in Table 1. An initial chest radiograph (Figure 1) revealed diffuse bilateral interstitial and alveolar opacities, and a subsequent non‐contrast chest computed tomography (CT) scan (Figure 2) showed diffuse airspace opacities with interlobular septal thickening and multiple centrilobular nodules, predominantly in the upper lobes. Further diagnostic studies were performed: ECG showed sinus rhythm with first‐degree AV block and prolonged QTc; echocardiography demonstrated preserved systolic function with diastolic dysfunction; venous Doppler revealed no deep vein thrombosis.

**TABLE 1:** Laboratory findings at presentation.

Parameter	Value	Reference range
Electrolytes and chemistry		
Sodium	133 mmol/L	135–145 mmol/L
Potassium	4.2 mmol/L	3.5–5.0 mmol/L
Chloride	107 mmol/L	98–107 mmol/L
BUN	16 mg/dL	7–20 mg/dL
Creatinine	0.98 mg/dL	0.6–1.2 mg/dL
eGFR	58 mL/min/1.73 m^2^	> 60 mL/min/1.73 m^2^
Anion gap	12 mmol/L	8–16 mmol/L
Random blood glucose	125 mg/dL	70–140 mg/dL
Liver function		
Alkaline phosphatase	93 U/L	40–150 U/L
Total bilirubin	0.7 mg/dL	0.1–1.2 mg/dL
Direct bilirubin	0.2 mg/dL	0.0–0.3 mg/dL
Albumin	2.6 g/dL	3.5–5.0 g/dL
ALT	12 U/L	7–56 U/L
AST	27 U/L	10–40 U/L
Complete blood count		
WBC	10.9 × 10^3^/μL	4.5–11.0 × 10^3^/μL
Neutrophils	85.4%	40%–75%
Absolute neutrophil count	9.3 × 10^3^/μL	1.8–7.7 × 10^3^/μL
Lymphocytes	6.3%	20%–45%
Hemoglobin	11.0 g/dL	12.0–16.0 g/dL
Hematocrit	34.0%	36%–46%
MCV	97.7 fL	80–100 fL
Platelets	392 × 10^3^/μL	150–450 × 10^3^/μL
Cardiac and inflammatory markers		
BNP	289.0 pg/mL	< 100 pg/mL
Procalcitonin	0.12 ng/mL	< 0.5 ng/mL
Troponin I	12 ng/L	< 14 ng/L
ESR	105 mm/h	0–30 mm/h
CRP	122.7 mg/L	< 10 mg/L
Endocrine studies		
HbA1c	5.6%	< 5.7%
TSH	0.35 μIU/mL	0.4–4.0 μIU/mL
Free T4	1.40 ng/dL	0.8–1.8 ng/dL
Arterial blood gas (on oxygen)		
pH	7.45	7.35–7.45
pCO_2_	33 mmHg	35–45 mmHg
pO_2_	42 mmHg	80–100 mmHg
HCO_3_	22.9 mmol/L	22–26 mmol/L
Base excess	−0.4 mmol/L	−2 to +2 mmol/L
O_2_ saturation	80.1%	> 95%

*Note:* Respiratory viral panel was negative for common respiratory pathogens including influenza, COVID‐19, RSV, metapneumovirus, rhinovirus, enterovirus, parainfluenza, *Bordetella pertussis* , and *Mycoplasma pneumoniae* .

### 2.4 Hospital Course

**FIGURE 3:**
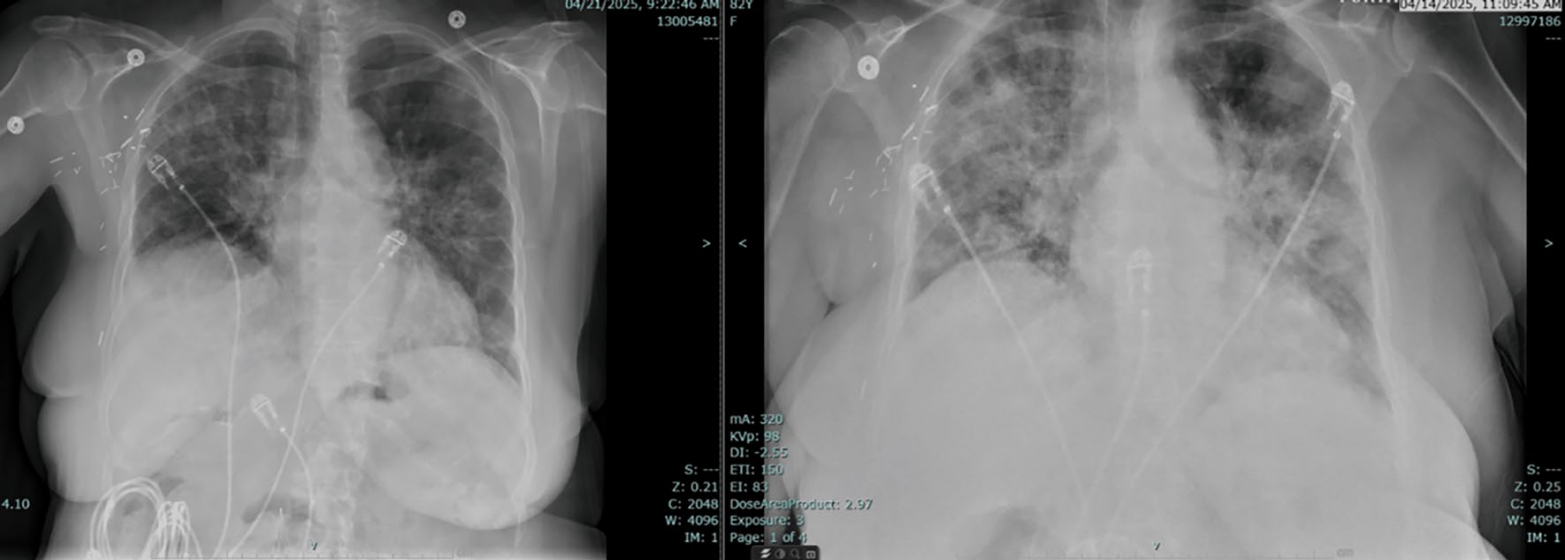
Follow‐up chest radiograph showing persistent diffuse opacities.

The patient developed asymptomatic bradycardia (HR high 50s) attributed to metoprolol (substituted for nebivolol), which was tapered from 200 mg to 50 mg daily. Oxygen requirements increased from nasal cannula. She was initiated on Bilevel positive airway pressure with FiO_2_ 40%; this modality was used as her prescribed CPAP device for obstructive sleep apnea was unavailable in the hospital, and its use was not necessitated by hypercapnia, which was absent. The patient subsequently stabilized on 8 L/min supplemental oxygen. Pulmonology consultation identified likely amiodarone‐induced lung injury. Treatment included amiodarone discontinuation and prednisone 40 mg daily. Trimethoprim‐sulfamethoxazole was added for Pneumocystis pneumonia prophylaxis. Cardiology consultation endorsed amiodarone discontinuation with consideration of flecainide for atrial fibrillation if needed, noting caution due to prolonged QTc interval. Follow‐up chest radiograph (Figure 3) showed persistent diffuse opacities without significant improvement.

### 2.5 Diagnosis and Disposition

The diagnosis was amiodarone‐induced pulmonary toxicity based on clinical presentation, medication history, laboratory findings, and imaging studies. Discharge oxygen requirements were 2 L/min at rest and 5 L/min with exertion. The patient was discharged home with supplemental oxygen and prednisone with a planned slow taper over several months. During subsequent follow‐up, her clinical condition continued to improve. When last seen in the cardiology office, her supplemental oxygen requirement had decreased to 2 L/min.

## 3 Discussion

This case report details the development of significant APT in an 82‐year‐old female after approximately 2 years of standard‐dose amiodarone therapy. The diagnosis was established based on her clinical presentation, characteristic radiographic findings, and the exclusion of other common etiologies. This case highlights the insidious nature of amiodarone toxicity, particularly in the elderly, the diagnostic utility of computed tomography (CT) of the chest, and the complex, multifactorial mechanisms potentially underlying its pathogenesis.

An alternative explanation for the acute deterioration could have been viral or secondary bacterial pneumonia, possibly progressing to ARDS. The elevated CRP and neutrophilia, along with the history of a viral prodrome, support this possibility. However, the negative viral panel, absence of fever, and improvement after drug withdrawal favored amiodarone‐induced toxicity [5, 6, 9].

Alternative causes of hypoxemia such as HFpEF, obstructive sleep apnea, and remote effects of prior breast cancer therapy were considered. The patient's preserved LV systolic function and normal BNP excluded overt heart failure. OSA was treated with CPAP but did not explain progressive hypoxemia. Prior breast cancer treatment was remote. BAL or biopsy was not pursued due to advanced age, severe hypoxemia, and procedural risk, consistent with standard practice in suspected APT when clinical and radiologic findings are strongly supportive [6, 10, 11, 12].

Amiodarone is a highly effective antiarrhythmic, yet its utility is constrained by a broad spectrum of potential adverse effects, with pulmonary toxicity being among the most severe and potentially life‐threatening [8, 9, 10, 13, 14]. The reported incidence of APT varies, with some sources indicating lung adverse effects in approximately 5% of treated patients [11], while a multicenter cohort study estimated an incidence of 1.9% in individuals on amiodarone for at least 90 days [6]. Other analyses suggest incidences between 1.6% and 6%, with lower figures potentially reflecting the use of reduced maintenance doses in more recent times [9, 15].

The clinical presentation of APT is often nonspecific, encompassing symptoms such as non‐productive cough, dyspnea, and exercise intolerance, which can progress to acute respiratory failure, making early recognition paramount [8, 9, 10, 11, 15]. In the patient described, the dyspnea had an insidious onset following a viral illness, which may have initially confounded the diagnostic picture. This diagnostic challenge is significant, as APT symptoms can mimic an exacerbation of heart failure, especially in elderly patients with comorbidities, thereby delaying appropriate management [15]. Furthermore, APT can be subclinical, detected incidentally on imaging or through a decline in the DLCO [10, 11].

Chest CT in our patient demonstrated diffuse interstitial and alveolar opacities with centrilobular nodules, consistent with recognized APT patterns [8, 11]. While radiologic findings can vary, ranging from interstitial pneumonitis to ARDS, early discontinuation of amiodarone and corticosteroid initiation remain the cornerstones of management [6, 10, 11, 12, 15, 16].

The pathogenesis is multifactorial, involving direct cytotoxicity and immune‐mediated injury, with phospholipidosis and foamy macrophages as common histologic features [6, 11, 14]. Elderly patients are particularly susceptible due to altered pharmacokinetics, cumulative dosing, and comorbidities [9, 10, 11]. Diagnosis is largely one of exclusion. BAL and biopsy were avoided here due to age and severe hypoxemia, but clinical and radiologic findings strongly supported APT [11, 12].

This case reinforces the importance of baseline and serial DLCO measurement, consideration of early imaging when respiratory symptoms develop, and timely corticosteroid initiation. Clinicians should maintain vigilance even with standard‐dose therapy in elderly patients [9, 10, 11, 15, 16].

## 4 Conclusion

In conclusion, this case highlights amiodarone‐induced pulmonary toxicity in an elderly woman on standard‐dose therapy. The nonspecific onset, confounding comorbidities, and characteristic radiology underscore the need for early suspicion, appropriate exclusion of alternative diagnoses, and prompt drug discontinuation with corticosteroids. Clinicians should adopt vigilant monitoring strategies to detect APT early.

## Author Contributions

**Bal Krishna Subedi:** conceptualization, methodology, validation, writing – original draft. **Naveen Gautam:** conceptualization, formal analysis, writing – original draft, writing – review and editing. **Anuja Upadhyay:** conceptualization, writing – review and editing. **Parikshit Chapagain:** conceptualization, validation, writing – review and editing. **Daniel Bitetto:** methodology, supervision, writing – review and editing.

## Ethics Statement

This case report was conducted in accordance with ethical principles.

## Consent

Written informed consent was obtained directly from the patient for the collection of clinical data and for the publication of this case report and any accompanying images. The patient was assured that all personal identifying information would be kept confidential.

## Conflicts of Interest

The authors declare no conflicts of interest.

## Data Availability

The data that support the findings of this study are available from the corresponding author, Naveen Gautam, upon reasonable request. The data are not publicly available due to privacy and ethical restrictions related to patient confidentiality.
